# An exploratory study into measuring the cortical bone thickness from CT in the presence of metal implants

**DOI:** 10.1007/s11548-017-1539-z

**Published:** 2017-02-23

**Authors:** Tristan Whitmarsh, Graham M. Treece, Andrew H. Gee, Kenneth E. S. Poole

**Affiliations:** 1 Department of Engineering, University of Cambridge, Trumpington Street, Cambridge, Cambridgeshire CB2 1PZ UK; 2 Department of Medicine, University of Cambridge, Cambridge, Cambridgeshire UK

**Keywords:** Computed tomography, Hip implants, Cortical thickness, Metal artefact removal

## Abstract

**Purpose:**

The aim of this study was to develop and evaluate a method for measuring the cortical bone thickness from computed tomography (CT) scans with metallic implants and to assess the benefits of metal artefact removal software.

**Methods:**

A previously validated technique based on the fitting of a cortical model was modified to also model metal structures when required. Cortical thickness measurements were taken over intact bone segments and compared with the corresponding contralateral bone segment. The evaluation dataset includes post-operative CT scans of a unipolar hemi-arthroplasty, a dynamic hip screw fixation, a bipolar hemi-arthroplasty, a fixation with cannulated screws and a total hip arthroplasty. All CT scans were analysed before and after processing with metal artefact removal software.

**Results:**

Cortical thickness validity and accuracy were improved through the use of a modified metalwork-optimised model and metal artefact removal software. For the proximal femoral segments of the aforementioned cases, the cortical thickness was measured with a mean absolute error of 0.55, 0.39, 0.46, 0.53 and 0.69 mm. The hemi-pelvis produced thickness errors of 0.51, 0.52, 0.52, 0.47 and 0.67 mm, respectively.

**Conclusions:**

The proposed method was shown to measure cortical bone thickness in the presence of metalwork at a sub-millimetre accuracy. This new technique might be helpful in assessing fracture healing near implants or fixation devices, and improve the evaluation of periprosthetic bone after hip replacement surgery.

## Introduction

Computed tomography (CT) has been shown to be a valuable tool in assessing the surgical outcome of hip fracture repair. Determining the patients’ long-term response to the surgical treatment by follow-up CT scans may also have considerable benefits in detecting pathological bone changes [1, 2].

Various complications can arise from surgical procedures to treat hip fractures, which will not all be immediately noticeable. Prosthetic replacement procedures can cause a redistribution of the load where the stress to the distal femur is now transferred through the metal stem, bypassing the femoral cortex. This stress shielding can subsequently result in bone atrophy at areas of low mechanical loading. Periprosthetic osteolysis after total hip arthroplasty, on the other hand, is believed to be a result of a chronic inflammatory reaction to particulate wear debris or the cement used in the fixation of the acetabular cup [3].

Osteolytic lesions can have serious consequences whereby extensive acetabular and femoral osteolysis may lead to pathological fractures which require complex revision surgery and are associated with significant morbidity. For an early diagnosis and preventative treatment, quality control after hip replacement surgery is therefore crucial.

Some studies have already shown CT to be a useful tool for detecting osteolysis following total hip arthroplasty, which were associated with an irregular thinning and discontinuity of the adjacent cortex [4]. Thus, measuring a thinning of the cortex from CT might be an early indicator of osteolysis and might indicate an increased risk of future fractures or other failures of the hip replacement.

**Fig. 1:**
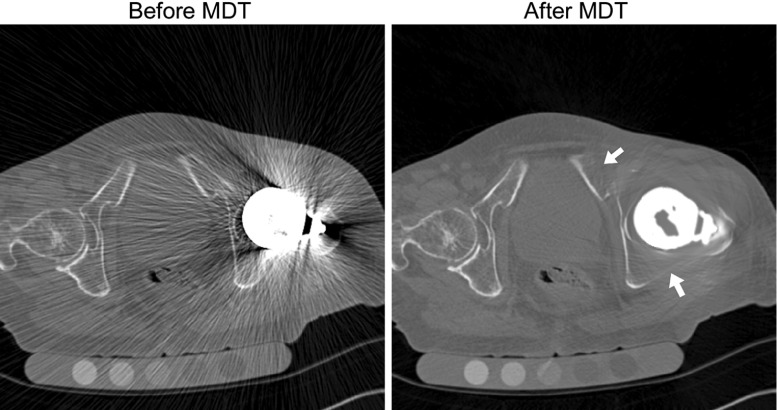
Axial CT slice with an Austin Moore hip implant before and after MDT, with the arrows indicating the fading or complete disappearance of the cortex

Some hip fractures require an internal or external fixation in order to stabilize the bone fragments and facilitate repair. In current clinical practice, fracture repair is largely assessed from planar radiographs. However, with recent advancements in medical image technologies, new quantitative fracture repair assessment techniques are being proposed which make full use of the spatial information available from CT scans [5–7]. These measurements are largely based on fracture gap bridging, but also on the extent of the callous formation and the degree of its mineralisation, which will be shown on CT as a thickening of the cortex. Thickness measurements from CT, however, are particularly difficult when metal hardware is present due to the high attenuation coefficient of metal.

In this study, we therefore propose a robust method for measuring the cortical bone thickness in the presence of metal hardware. Adaptations to a previously developed cortical thickness measurement technique are presented to account for the high attenuation values of metal. The evaluation compares cortical thickness measurements with the contralateral bone segment and includes an assessment of the benefits of metal artefact removal software.

## Material and methods

### Study population

Post-operative hip CT scans were collected from five patients, each of whom underwent a different hip repair or replacement procedure. These include a unipolar hemi-arthroplasty using an Austin Moore prosthesis (female, 77 years), a dynamic hip screw placement (female, 78 years), a bipolar hemi-arthroplasty (female, 83 years), a placement of three cannulated screws (female, 78 years) and a total hip arthroplasty (male, 71 years). The CT scans are part of a retrospective study which received approval by the institutional Research Ethics Committee (LREC 99/076 and LREC 04/0108). Volume renderings of the post-operative fractured hips are provided in Fig. 3.

## Imaging protocol

All images were acquired on a Siemens Sensation 16 Slice CT system (Siemens Medical Solutions, Forchheim, Germany). The scanning parameters were as follows: tube current, 98–156 mA; exposure, 73–117 mAs; tube voltage, 120 kV; slice thickness 1 mm; and pixel spacing 0.59$\backslash$0.59 mm.

### Metal artefact removal

Metal hardware results in streak artefacts in the CT scan images due to beam hardening effects as well as noise from X-ray scatter [8]. The extent of the artefacts depend on the kVp and mAs used in the CT scan acquisition, as well as the position of the metal structure in the CT scanner. Artefacts can severely degrade the CT images, and many methods have already been proposed to remove them. In this work, we use the metal deletion technique (MDT) software^1^ described in [9], which has been shown to successfully remove artefacts resulting from beam hardening, Poisson noise and patient motion. An example of this is provided in Fig. 1. Standard settings were used with a metal cut-off point of 3000 HU.

### Cortical thickness measurements

This work builds further on a previously published cortical thickness measurement technique which measures the cortical thickness for typical cortices with a mean (± SD) error of 0.12 ± 0.39 mm [10]. This technique, which has been implemented in the Stradwin^2^ software tool, measures the cortical thickness by sampling the CT values along a line perpendicular to the bone surface. A blurred model of the cortex, described as a combination of step functions, is subsequently fitted to the data samples, which results in a measure of the cortical thickness [10–12]. Here the parameters of the step functions, as well as the extent of the blur, are optimised in order to minimise the difference between the blurred model and the data samples.

This technique requires a mesh to be constructed of the bone surface whereby the vertices define the locations of the cortical measurements. While the mesh vertices define an initial location of the periosteal bone surface, a more accurate location is determined by finding the nearest inflection point. An estimate of the cortical endpoint is then found by searching for the next inflection point. In an optimisation process, the start and end location as well as the endocortical, cortical and soft tissue CT values are then found such that the blurred model of the cortex best fits the real data samples. This further refines the estimate of the cortical thickness.

Cortical bone is a highly irregular structure with pores, outcrops and connected trabecular lattices which can all negatively affect the accuracy of the cortical thickness measurements. Similar effects occur when the femoral head comes in close proximity to the acetabulum. In order to prevent any inner or outer bone structures from being incorporated into the cortex or disrupting soft tissue or trabecular values, we now also model an additional inner and outer bone structure. The model now optionally includes the start and end position of an inner and outer step function, which is again initialised by the inflection points. As described previously, the smoothed step function is then fitted to the data while optimising also the locations of the inner and outer bone structures. If the model fails to fit, then the software attempts to fit the model without the inner structure. If the model still does not fit the data, then it will try to fit the model without both the inner and outer structure.

### Metal modelling

While metal artefact removal software reliably removes streak artefacts, the regions in the CT scans that contain metal will have CT values significantly greater than those of soft tissue or bone. The Hounsfield Unit (HU) of titanium is generally around 8000 HU while that of stainless steel is around 13000 HU, although this is largely dependent on the kVp used. Furthermore, due to the 12-bit precision of most CT devices, commonly allowing for a range of between -1024 and 3071 HU, metal CT values are clamped at the maximum value. These inconsistent CT values will significantly disrupt the thickness measurements if not handled appropriately. Modifications are therefore made to the process described above to model a metal structure in place of an inner or outer bone structure where appropriate.

If, upon searching the inner or outer bone structure, metal is found (as identified by having a HU greater than 3000), a metal step function is defined instead. This step function includes the start point of the metal structure, which is initialised at the location where the CT value is greater than 3000. No endpoint is defined since samples are not considered beyond the metal start point. Considering metal has varying CT values, depending on the scanner settings and material properties, the metal CT value level is also left to be optimised.

**Fig. 2:**
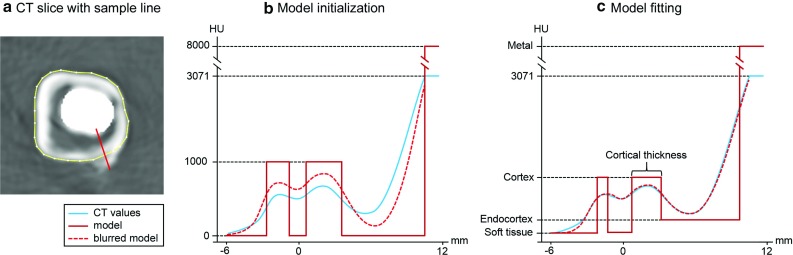
Schematic representation of the model fitting process with an outer bone structure from the linea aspera and an inner metal structure from the stem of an Austin Moore hip implant. **a** A section of an axial CT slice just below the lesser trochanter with the manually defined bone contour (*yellow*) and the sample line (*red*). **b** A plot of real data samples (*blue*), the initialisation of the model of the cortex (*red*) and the smoothed model (*red dashed*) before fitting the model to the data samples. **c** The model fitted to the data such that the smoothed model optimally matches the data samples, resulting in a measurement for the cortical thickness

In Fig. 2 we show the model fitting process with an outer bone structure as described in “Cortical thickness measurements” section and an inner metal structure.

### Analysis

**Fig. 3:**
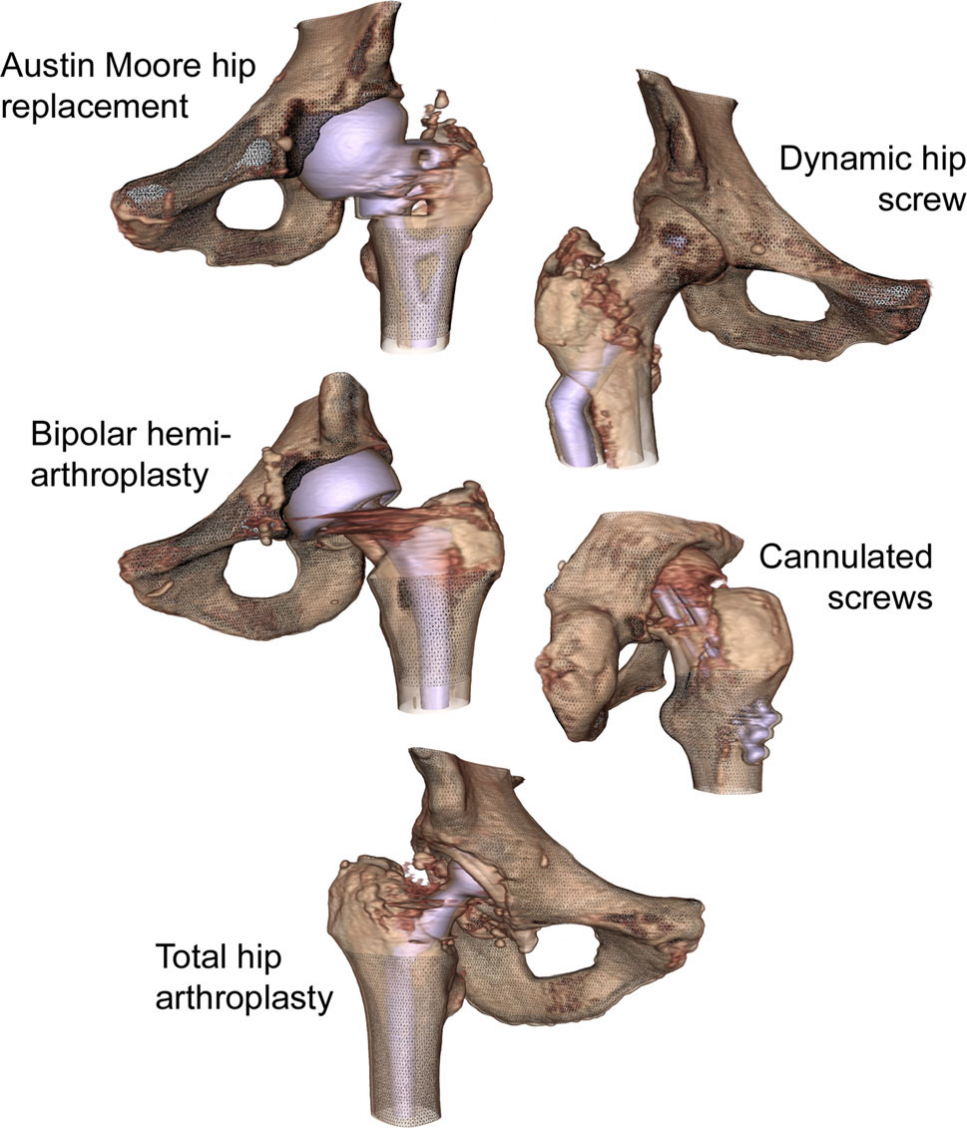
CT scan volume renderings of the five surgically treated hips used in this study with five different types of metallic implants. Mesh structures are shown along with the volume renderings whereby cortical thickness measurements are taken at all the mesh vertices

Bone regions were meshed to incorporate large portions of the bone segments, while minimising interferences from the fracture lines (Fig. 3). By measuring the thickness at each vertex, we can display a colour coded map of the thickness measurements (cortical thickness map). The cortical thickness maps were compared with their contralateral intact counterpart, of which the CT scans were processed with MDT to remove any streak artefacts emanating from the metal. A one-to-one correspondence was established using the software tool wxRegSurf,^3^ which maps the cortical thickness measurements from the contralateral intact bone to the corresponding locations on the mesh of the metalwork-affected bone, or in some cases, the other way around. This gives us cortical thickness measurements at corresponding locations on both bone segments and will allow us to assess the accuracy of the measurements quantitatively.

### Statistics

All of the analyses were carried out in MATLAB version R2013b (The MathWorks Inc., Natick, MA, USA). Mean and absolute differences are given for cortical thickness measurements when modelling only inner and outer bone structure and also with an optional modelling of metal structures. In addition, the Pearson correlation coefficients are computed over the pairs of thickness measurements to assess the linear relationship between the thickness measurements in the fractured and the contralateral bone. Considering the blurring in the CT data is dramatically affected by the artefact removal technique which might negatively affect the thickness measurements, results are presented both with and without MDT software applied to the CT scans. Significances of the signed measurement errors were assessed by Student’s *T* tests. The significances of the differences for modelling metal, applying MDT or both, compared to when neither metal modelling nor MDT was applied, were assessed by paired *T* tests on the absolute errors.

Some measurements do not result in a valid thickness where the optimiser fails to fit the cortical model to the data samples. The aggregate results, therefore, incorporate only the sample points where all four types, as well as the contralateral bone, have a valid thickness. However, an important improvement of the metal modelling technique is the fact that more samples produce valid measurements. Thus, also the number of successful thickness measurements is provided.

**Table 1:** The error values of the cortical thickness measurements with respect to the contralateral bone segment

	Proximal femur segment				Hemi-pelvis			
	Sample count	Mean diff ± SD	Mean abs	PCC	Sample count	Mean diff ± SD	Mean abs	PCC
*Austin Moore implant*		*n* = 1946				*n* = 12,729		
No metal modelling	2252	$-0.40 \pm 1.26^{*}$	0.87	0.607	13,794	$-0.06 \pm 0.95^{*}$	0.57	0.413
Metal modelling	2850	$-0.18 \pm 1.16^{*}$	$0.78^{\dagger }$	0.695	15,021	$-0.04 \pm 0.94^{*}$	0.57$^{\dagger }$	0.421
No metal modelling with MDT	2319	$-0.18 \pm 1.09^{*}$	0.67$^{\dagger }$	0.659	13,609	$-0.12 \pm 0.88^{*}$	0.51$^{\dagger }$	0.464
Metal modelling with MDT	2965	$-0.01 \pm 0.89$	$0.55^{\dagger }$	0.783	14,887	$-0.11 \pm 0.87^{*}$	0.51$^{\dagger }$	0.465
*Dynamic hip screw*		*n* = 2884				*n* = 13,397		
No metal modelling	3417	$0.16 \pm 0.68^{*}$	0.46	0.586	14,330	$-0.00 \pm 0.78$	0.53	0.300
Metal modelling	3572	$0.18 \pm 0.66^{*}$	0.46	0.604	14,330	$-0.00 \pm 0.78$	0.53	0.299
No metal modelling with MDT	3522	$0.15 \pm 0.54^{*}$	$0.38^{\dagger }$	0.696	14,697	$0.06 \pm 0.77^{*}$	0.52$^{\dagger }$	0.310
Metal modelling with MDT	3669	$0.17 \pm 0.54^{*}$	$0.39^{\dagger }$	0.697	14,695	$0.06 \pm 0.77^{*}$	0.52$^{\dagger }$	0.310
*Bipolar hemi-arthroplasty*		*n* = 2535				*n* = 12,243		
No metal modelling	2599	$0.11 \pm 0.91^{*}$	0.57	0.524	13,202	$-0.03 \pm 0.88^{*}$	0.57	0.429
Metal modelling	2921	$0.13 \pm 0.89^{*}$	$0.55^{\dagger }$	0.546	14,880	$-0.02 \pm 0.87^{*}$	0.56	0.437
No metal modelling with MDT	2655	$-0.24 \pm 0.68^{*}$	$0.47^{\dagger }$	0.691	13,071	$-0.03 \pm 0.82^{*}$	0.53$^{\dagger }$	0.493
Metal modelling with MDT	2983	$-0.23 \pm 0.67^{*}$	$0.46^{\dagger }$	0.700	14,607	$-0.03 \pm 0.81^{*}$	0.52$^{\dagger }$	0.496
*Cannulated screws*		*n* = 2085				*n* = 12,283		
No metal modelling	2164	$-0.02 \pm 0.97$	0.58	0.668	12,564	$-0.00 \pm 0.75$	0.47	0.504
Metal modelling	2181	$-0.01 \pm 0.96$	0.57	0.671	12,564	$-0.00 \pm 0.75$	0.47	0.504
No metal modelling with MDT	2166	$-0.27 \pm 0.74^{*}$	$0.53^{\dagger }$	0.776	12,578	$-0.03 \pm 0.77^{*}$	0.47	0.494
Metal modelling with MDT	2184	$-0.26 \pm 0.74^{*}$	$0.53^{\dagger }$	0.774	12,581	$-0.03 \pm 0.76^{*}$	0.47	0.496
*Total hip arthroplasty*		*n* = 4199				*n* = 18,970		
No metal modelling	4410	$-0.68 \pm 1.63^{*}$	1.09	0.639	19,685	$-0.04 \pm 1.07^{*}$	0.68	0.622
Metal modelling	5068	$-0.40 \pm 1.39^{*}$	$0.93^{\dagger }$	0.759	19,713	$-0.04 \pm 1.07^{*}$	0.68	0.623
No metal modelling with MDT	4555	$-0.73 \pm 1.56^{*}$	$1.05^{\dagger }$	0.675	19,688	$-0.01 \pm 1.05$	$0.67^{\dagger }$	0.633
Metal modelling with MDT	5094	$-0.28 \pm 0.96^{*}$	$0.69^{\dagger }$	0.891	19,691	$-0.01 \pm 1.05$	$0.67^{\dagger }$	0.633

*Mean diff* mean difference calculated by subtracting the cortical map of the contralateral hip from the cortical map of the hip with metalwork, *Mean abs* mean absolute difference, *SD* standard deviation, *PCC* Pearson correlation coefficient, *MDT* metal deletion technique.

$^{*}~$Significant (*p* < 0.05) assessed by a Student’s *T* test. $^{\dagger }~$Significantly (*p* < 0.05) different with respect to the absolute errors without metal modelling or MDT, assessed by a paired *T* test

**Fig. 4:**
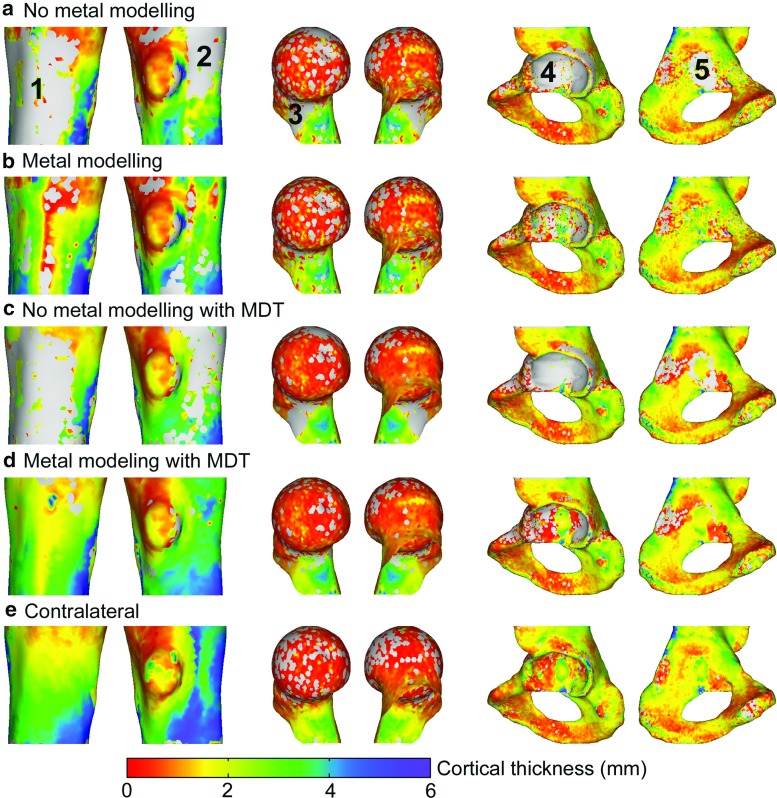
Cortical thickness maps of the proximal femur segment from the Austin Moore hip replacement, the femoral head and neck with dynamic hip screws and the hemi-pelvis from the bipolar hemi-arthroplasty. Here we show the segment of the bone with the metal implant when only modelling inner and outer bone structures (**a**), when modelling also metal structures (**b**), with metal artefact removal applied but no metal modelling (**c**) and with both metal modelling and metal artefact removal (**d**). Also the cortical thickness map of the corresponding contralateral bone segment is shown (**e**) which is defined on the same mesh to allow for a point-wise comparison (described in “Cortical thickness measurements” section). The *grey regions* indicate no valid thickness measurements

## Results

Quantitative results are presented in Table 1. For the shaft sections, mean absolute differences were consistently decreased when also modelling metal structures compared to only modelling inner and outer bone structures, both with and without metal artefact removal. Applying both the metal modelling and MDT provides the smallest mean absolute error, which are statistically significantly different (*p* < 0.05) with respect to no metal modelling and no MDT applied. These improvements are also reflected by the Pearson correlation coefficients.

For the hemi-pelvis, we can see no improvements in the cortical thickness measurements when modelling metal since the metal structures are of sufficient distance from the cortex or, in the case of the Austin Moore hip replacement and bipolar hemi-arthroplasty, the acetabular cup prevents measurements at the acetabulum altogether.

Examining the mean differences of all bone segments, we find some cases with a significant over or under estimation of the thickness. The bias, however, is relatively small, ranging from an overestimation of 0.17 mm for the femoral segment with a dynamic hip screw to an underestimation of −0.28 mm for the femoral segment of the total hip arthroplasty.

For all bone segments, apart from the shaft section of the total hip arthroplasty and the pelvic segment of the patient with cannulated screws, we can see that the MDT improves the measurement more than modelling metal alone. However, not of lesser importance is the fact that metal modelling, in most cases, provides more successful measurements than without. In particular at the femoral head and neck with the dynamic hip screw, modelling metal does not produce a reduced error, but does result in considerably more measurements, which can also be seen in Fig. 4 (region 3).

Figure 4 shows the cortical thickness maps of the femoral segment with the Austin Moore hip replacement, the femoral head and neck with a dynamic hip screw and the hemi-pelvis of the bipolar hemi-arthroplasty. Although not representative for the other bone structures, these three cases do show some interesting effects resulting from the methods used. We can see that for all three bone sections, not modelling metal results in a large region near the metal with no valid measurements, shown in grey (Fig. 4, region 1, 2, 3 and 5). Thus, thickness measurements appear to be greatly affected by metal, even when metal artefact removal is applied first. Although including a model of the metal results in more successful measurements, without metal artefact removal these measurements appear to remain noisy.

At the cortex of the acetabulum, few thickness measures can be obtained, even with metal modelling and MDT. Although the subchondral bone plate of the acetabulum is preserved in a hemi-arthroplasty procedure, the acetabular cup rests against the acetabulum and, at this close proximity, largely obscures the cortex. Furthermore, metal artefact removal software is not successful in close proximity to large metal objects and effectively removes the contrast in the reconstructed CT image, making measurements of the cortex virtually impossible (Fig. 4, region 4). Also, in other regions where metal produces large streak artefacts, MDT removes the cortex almost entirely (Fig. 1). Unfortunately, without metal artefact removal, the thickness measurements are likely faulty due to these streak artefacts.

Altogether we can see that modelling the inner and outer metal structures results in the increase in successful measurements with an overall improved accuracy, which is further enhanced by an initial metal artefact removal process applied to the CT scan.

## Discussion

The results indicate that the proposed modifications to the cortical thickness measurement technique, whereby metal structures are effectively modelled, improved the success rate and accuracy of the measurements. Applying metal artefact removal software to the CT scans further enhanced the accuracy of the measurements.

Some previous studies have already assessed the accuracy of bone thickness measurements from CT imaging systems near dental implants. In [13], the authors report an underestimation of the cortical bone thickness on two cone beam CT systems. Wang et al. [14] report a mean (standard deviation) difference between radiological and histological measurements of −0.22 (0.77) mm, thus also underestimating cortical thickness in cone beam CT. Conversely, Gerlach et al. [15] report an overestimation of the cortical thickness from cone beam CT compared to a histological evaluation. Furthermore, in [16] metal artefacts were shown to increase bone thickness measurements in multi-slice CT and cone beam CT by 5 and 6%, respectively, although the differences when comparing the measurements with and without metal artefacts were not significant. In these studies, however, thickness measurements are performed manually. These measurements are therefore affected by more factors than the imaging modality alone and will not reach the sub-voxel level of accuracy of the de-convolution method presented here. In our study, we see a decrease in mean error when metal artefact removal is used, while over- or underestimations of the thickness measurements remain relatively small considering the spatial resolution of the clinical CT scans.

Although metal artefact removal software has been shown to successfully remove streak artefacts, the effects are limited in close proximity to the metalwork. In the Austin Moore hip replacement and the bipolar hemi-arthroplasty, the cortex disappears where regions are only visible from limited angles due to the obstruction of metal. Thus, at the acetabulum, few reliable samples of the thickness can be made. Furthermore, the acetabular cup placement within a total hip arthroplasty requires acetabular reaming, which partially or completely removes the acetabular cortex. However, osteolysis does not necessarily occur near the point of contact between the artificial hip and the acetabulum and can appear more exterior to the cup placement. Indeed Claus et al. [17] have already shown that computed tomography combined with metal artefact removal can become a useful tool in diagnosing and monitoring periacetabular osteolysis associated with implants. Thus, the measurement of the cortical thickness over the entire bone region might still be of great benefit in identifying adverse effects of prosthetic replacement surgery.

The most common measure in fracture healing from plain radiographs involves measuring the bridging of the fracture site by calcifying callus. In [18] a callus index is defined as “the ratio of the maximum callus diameter to bone diameter at the same level as the callus”. This article reports that, in patients with a tibial fracture treated by an intramedullary nail, this index exceeded 1.45 when measured from anteroposterior radiographs. When considering an average tibial diameter, this translates to a callous thickness well above the error reported in our study. Thus, a longitudinal analysis of the cortical thickness change might be a useful tool in fracture healing assessment and indicating further treatment requirements.

Some limitations of this study have to be noted. While a comparison was made with the contralateral bone, this might not be the best reference. Symmetry studies have previously shown a strong correlation between the left and right femur in dual-energy X-ray absorptiometry-based bone mineral density measurements [19], and the results of a recent study on densitometric and geometric measurements as well as axial and bending rigidities of the left and right femur [20] further support the use of the contralateral femur as an intra-subject control. However, differences do exist due to local discrepancies, such as osteophytes, which are not symmetric in nature. Reported errors in this study are hence conservative and include errors due to bilateral asymmetry and measurement precision.

A conclusive evaluation of this new technology requires a dataset of pre and post-operative CT scans, which will not include any errors induced by bilateral asymmetry. However, this may still include an error from damage done to the bone during surgery. Therefore, for an ideal evaluation we propose an ex vivo experimental study on hip bone specimens with pre and post-operative clinical CT scans.

To conclude, we have presented a method to accurately and reliably measure the cortical thickness over the bone surface in the presence of metal structures. This technique may lead to a tool for assessing the progress of fracture healing or to assess the long-term effects of reconstructive surgery and prosthetic replacement surgery.

## Footnotes

1
http://www.revisionrads.com.

2
http://mi.eng.cam.ac.uk/~rwp/stradwin/.

3
http://mi.eng.cam.ac.uk/~ahg/wxRegSurf/.
